# Identifying patients at risk in revision arthroplasty: a comprehensive single-centre analysis

**DOI:** 10.1186/s13018-025-06625-y

**Published:** 2026-01-15

**Authors:** Matthias Wolf, Burkhard Lehner, Andreas Geisbüsch, Christian Merle, Tilman Walker, Julian Deisenhofer

**Affiliations:** 1 Universitätsklinikum Heidelberg, Orthopädische Universitätsklinik, Schlierbacher Landstraße 200a, 69118 Heidelberg, Germany; 2 Diakonie-Klinikum Stuttgart, Rosenbergstraße 38, 70176 Stuttgart, Germany

**Keywords:** Arthroplasty, Hip, Knee, Complication, Comorbidity, Surgical procedures, Emergency, Perioperative care

## Abstract

**Background:**

The demand for revision hip and knee arthroplasty (rTHA/rTKA) is increasing, while they continue to be associated with greater perioperative risks, higher resource demands, and greater variability in outcomes compared with primary procedures. Identifying precise risk factors is essential for effective perioperative management and resource planning.

**Methods:**

A retrospective analysis of 2,123 revision total hip (rTHA, *n* = 1,301) and knee arthroplasties (rTKA, *n* = 822) performed from 2010 to 2019 at a tertiary German centre was conducted. Adverse events (AE), length of hospital stay (LOS), and predictors including age, Elixhauser Comorbidity Index (EI), joint type, and indication were analysed using multivariate regression models.

**Results:**

The overall AE rate was 13.1%, significantly higher in rTHA than in rTKA (12.6% vs. 8.8%; *p* = 0.008), particularly for infections and mechanical complications. Mean LOS was 19 ± 14 days. Infection, age, EI, joint type, dislocation, and periprosthetic fracture independently predicted AE and LOS. Infection was the strongest predictor overall (AE: OR 5.4; LOS: Coefficient 1.6), with periprosthetic fractures being highly predictive in rTKA (OR 9.8).

**Conclusions:**

Infection (in all revisions), periprosthetic fractures (in rTKA), advanced age, and high comorbidity burden were critical determinants of perioperative adverse events and hospital utilisation. Focused perioperative care strategies targeting these risk groups are essential to mitigate adverse outcomes and optimise healthcare resources.

**Supplementary Information:**

The online version contains supplementary material available at 10.1186/s13018-025-06625-y.

## Background

The growing prevalence of total hip and knee arthroplasties has led to an increasing number of revision procedures. Compared to primary arthroplasty, revision total hip arthroplasty (rTHA) and revision total knee arthroplasty (rTKA) are more complex, technically demanding, and associated with significantly higher perioperative risks [1], including complications, prolonged hospital stays, and increased costs. Previous studies have identified surgical factors such as comorbidities, procedural complexity and surgeon experience as contributors to adverse events (AE) [2–5].

Age and comorbidity burden are well-established preoperative risk factors [4], while the Elixhauser Comorbidity Index (EI), based on a set of Elixhauser Comorbidities (EC), remains one of the most robust tools for predicting postoperative outcomes [6]. Despite these insights, the interaction of surgical indications and underlying risk factors is insufficiently understood, in particular how these factors combine to influence the likelihood of AE across indications and joint types remains challenging to determine.

This study retrospectively reviews a decade of revision arthroplasty procedures performed at a large tertiary academic centre in Germany. Within this high-risk cohort our primary aim was (1) to determine the incidence of in-hospital AE and the duration of hospital stay (LOS), across joints and indications; (2) to evaluate the association between risk factors, joint, indication and AE, as well as LOS; and (3) to identify independent predictors for AE and prolonged LOS to detect “patients-at-risk” who may benefit from closer perioperative care and possibly guide resource allocation.

## Methods

We conducted a retrospective cohort study of all patients who underwent revision total hip or knee arthroplasty (rTHA/rTKA) at a German tertiary academic hospital. The study period spanned from 1 January 2010 to 31 December 2019.

All patients who underwent any form of revision arthroplasty (hardware revision, including inlay replacement) of the hip or knee were included, regardless of underlying indication. Cases were identified from hospital administrative databases using diagnosis codes (ICD-10-GM) and procedure codes (OPS, German adaptation of ICD-10-PCS) and subsequent individual review of patient charts. To improve data homogeneity, rare indications occurring in fewer than 1% of all cases were excluded from further analysis. All cases with complete indication, procedure and AE data were included; LOS data were reported when available.

Comorbidities were quantified using the Elixhauser Comorbidities (EC, Elixhauser et al. (1998), which are well-documented surgical outcome predictors [6, 7] and the subsequently derived Elixhauser Index (EI) in its modification by Walraven et al. (2009), representing the overall comorbidity burden [8].

The primary outcome was defined as the occurrence of any adverse event (AE) during the initial hospital stay. For patients discharged within 30 days of the index procedure, adverse events during any readmission within this 30-day perioperative period were also included. Adverse events were identified from ICD/OPS codes and associated surgical commentary and were classified as either surgical (e.g. infection, haematoma, wound problems, vascular injury) or medical (e.g. acute renal failure, myocardial infarction, pulmonary embolism, stroke) and in-hospital amputation and mortality. This methodology included all relevant AE of Clavien-Dindo Grade ≥ 3 [9]. A systematic classification process is illustrated in Appendix A.

The secondary outcome was LOS, calculated in days from the date of admission to the date of discharge for the index hospitalisation.

Descriptive statistics summarised baseline demographics, surgical indications, joint type and comorbidity burden, both assessed as comorbidity count (number of EC) and EI. Chi-square or Fisher’s exact tests were used to assess the associations between surgical indication, joint type, comorbidity burden and AE, as well as LOS.

Multivariate logistic regression was applied to identify independent predictors of AE and generalised linear models were used to determine associations with LOS, both adjusting for comorbidity burden and age. Subsequent analyses were stratified by joint type and compared effects between hips and knees, identifying significant differences in AE risk and LOS. P-values, coefficients and odds ratios (OR) with 95% confidence intervals (CI) were reported. Statistical significance was defined as *p* < 0.05.

## Results

A total of 2,218 revision arthroplasty procedures were performed between 2010 and 2019. Five rare indications (material fracture, instability, material failure, tumour progression, and other), each representing fewer than 1% of cases (< 21 cases), were excluded. The final study cohort included 2,123 cases, of which 1,301 were revision total hip arthroplasties (rTHA, 61.3%) and 822 were revision total knee arthroplasties (rTKA, 38.7%). Of these, 115/822 (14.0%) involved uni-compartimental knee arthroplasty (UKA) conversion and 13 isolated inlay replacements in UKA. The mean patient age was 67.4 ± 14 years, while rTHA patients (68,9 ± 12.6) were older than rTKA patients (64.9 ± 15.8) with a generally high comorbidity burden (mean Elixhauser Index [EI] 4.9 ± 7.4).

A total of 678 subsequent revision surgeries were performed during the index hospitalisation (range, 0–4 per patient). Intensive care was required in 371 cases (17.5%), and blood transfusions were administered in 1,339 cases (63.1%). In total, 279 AE were observed in 223 patients; of these, 101 (33.6%) were medical and 171 (61.3%) were surgical. See Appendix C for details. There were 14 amputations (0.7%) and 27 in-hospital deaths (1.3%). There was a small paediatric subpopulation of 15 patients (age 9–17, no ACO within the subgroup). The mean length of hospital stay (LOS) was 19 ± 14 days (Table 1), with LOS data missing in 64 cases (3%).

**Table 1:** Descriptives and outcomes parameters

Descriptives		Count / Mean (± SD)	Frequency (%) / Median [IQR]
Total cohort		2123	100
Age		67.4 (± 14.0)	9–95
Elixhauser Index		4.9 (± 7.4)	3 [0–8]
Revision hip arthroplasty		1301	61.3
Revision knee arthroplasty		822	38.7
Adverse Event		279 (223 cases)	13.1 (10.5 of cases)
Amputation		14	0.7
Death		27	1.3
Length of stay (LOS, days)		19,0 (± 14.0)	15 [11–22]
ICU treatment (cases)		371	17.5
Blood transfusions		1339	63.1
Cell salvage		444	20.1
Subsequent revisions per case	Total cases	2123	100
	1	393	18.5
	2	72	3.4
	3	27	1.3
	4	15	0.7

IQR – Interquartile Range; ICU – intensive care unit. Patients could experience multiple adverse events, their incidence is expressed both as the proportion of all AE (13.1%) and as the proportion of cases in which at least one AE occurred (10.5%). The table summarises descriptives and outcome parameters

**Fig. 1:**
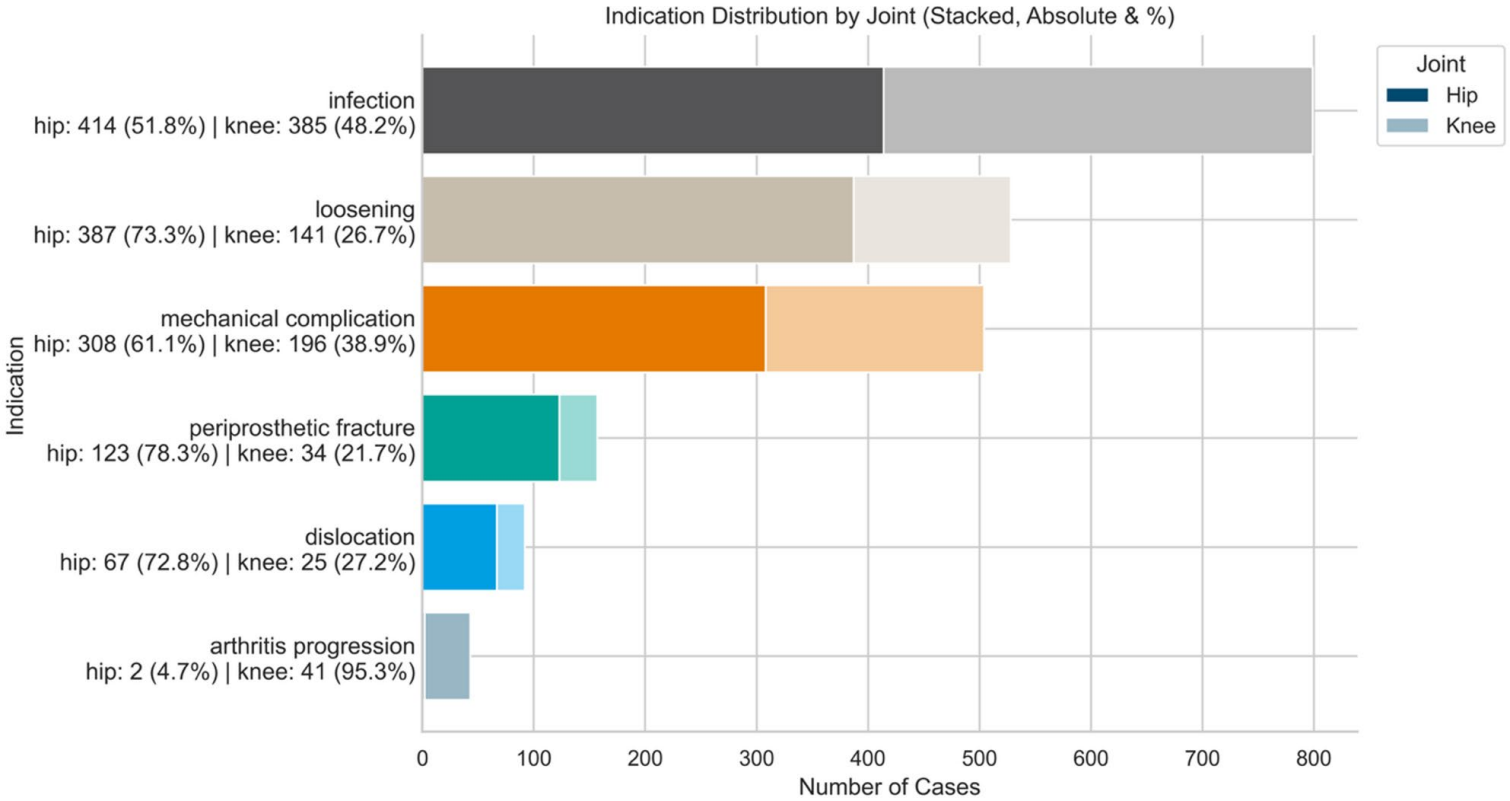
Distribution of surgical indications.N - number of cases, % - percentage. Stacked bar plot showing indication distribution for revision arthroplasty by joint (hip vs. knee). Bar segments indicate absolute case numbers and within-indication percentages for each joint. Infection was the most common indication overall

The most frequent indication for revision surgery was infection (799 cases, 37.7%), followed by loosening (528 cases, 24.9%), mechanical complication (504 cases, 23.7%), periprosthetic fracture (157 cases, 7.4%), dislocation (92 cases, 4.3%) and arthritis progression (43 cases, 2.0%). While infection was equally distributed among joints, dislocation, periprosthetic fracture, mechanical complication and loosening were more prevalent in hip revisions. Arthritis progression was almost exclusively seen in knee revisions, reflecting the conversion from unicompartmental knee arthroplasty to total knee arthroplasty (Fig. 1).

**Fig. 2:**
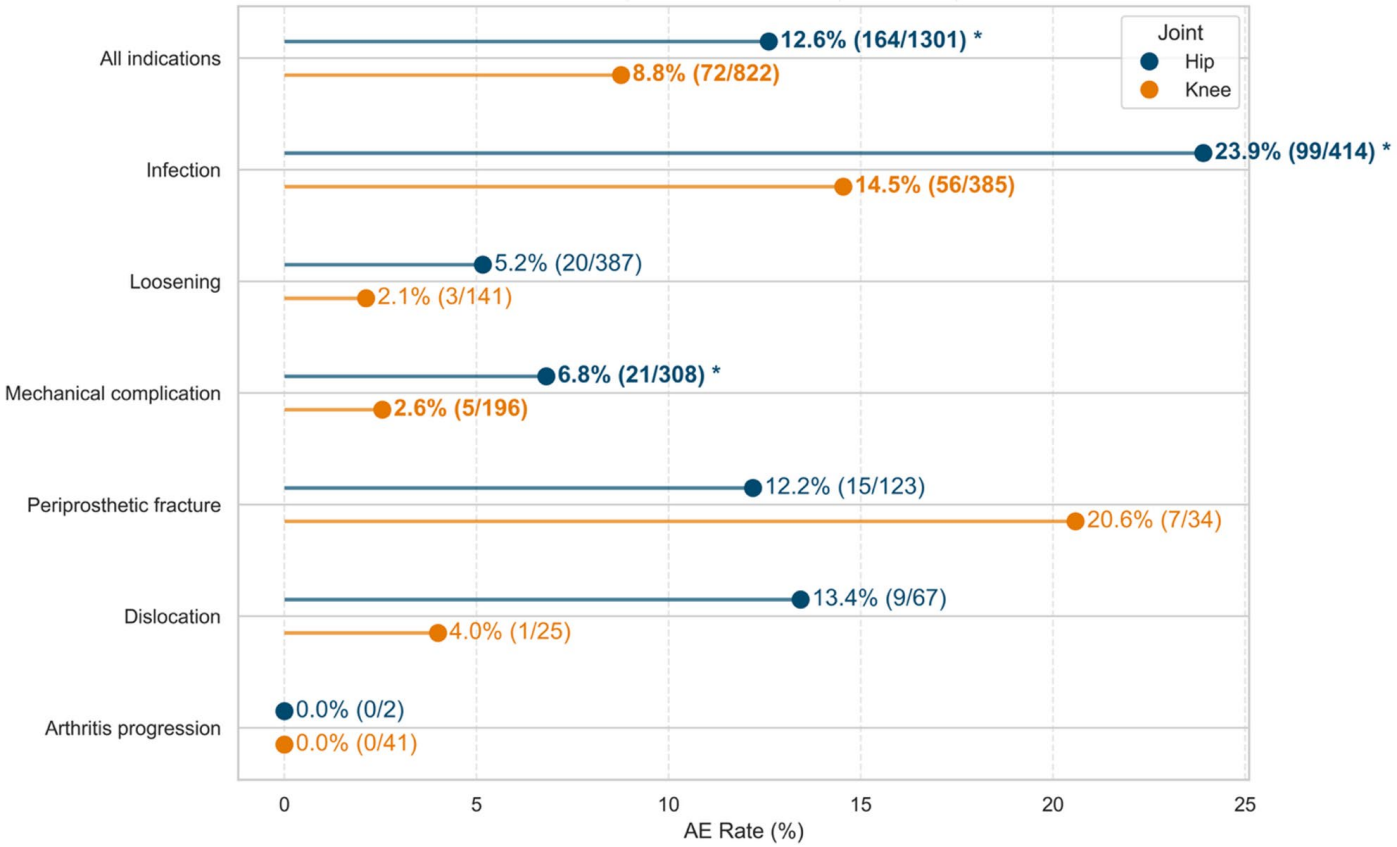
Adverse event rate by joint and indication. AE – Adverse Event. Lollipop plot showing AE rates stratified by indication and joint. Indications (or overall) with significant differences in AE rates between hip and knee are marked with asterisks (*p < 0.05)

The overall AE rate was significantly higher in hip revisions than in knee revisions (12.6% [164/1,301] vs. 8.8% [72/822], *p* = 0.008). Stratification by joint and surgical indication demonstrated higher AE rates in hips compared to knees for both infection (23.9% [99/414] vs. 14.5% [56/385]; *p* = 0.001) and mechanical complications (6.8% [21/308] vs. 2.6% [5/196]; *p* = 0.035). Trends towards increased AE in hips were also observed for dislocation and loosening, though these did not reach statistical significance. Periprosthetic fracture was the only indication with a non-significant trend towards higher AE rates in knees (Fig. 2).

**Fig. 3:**
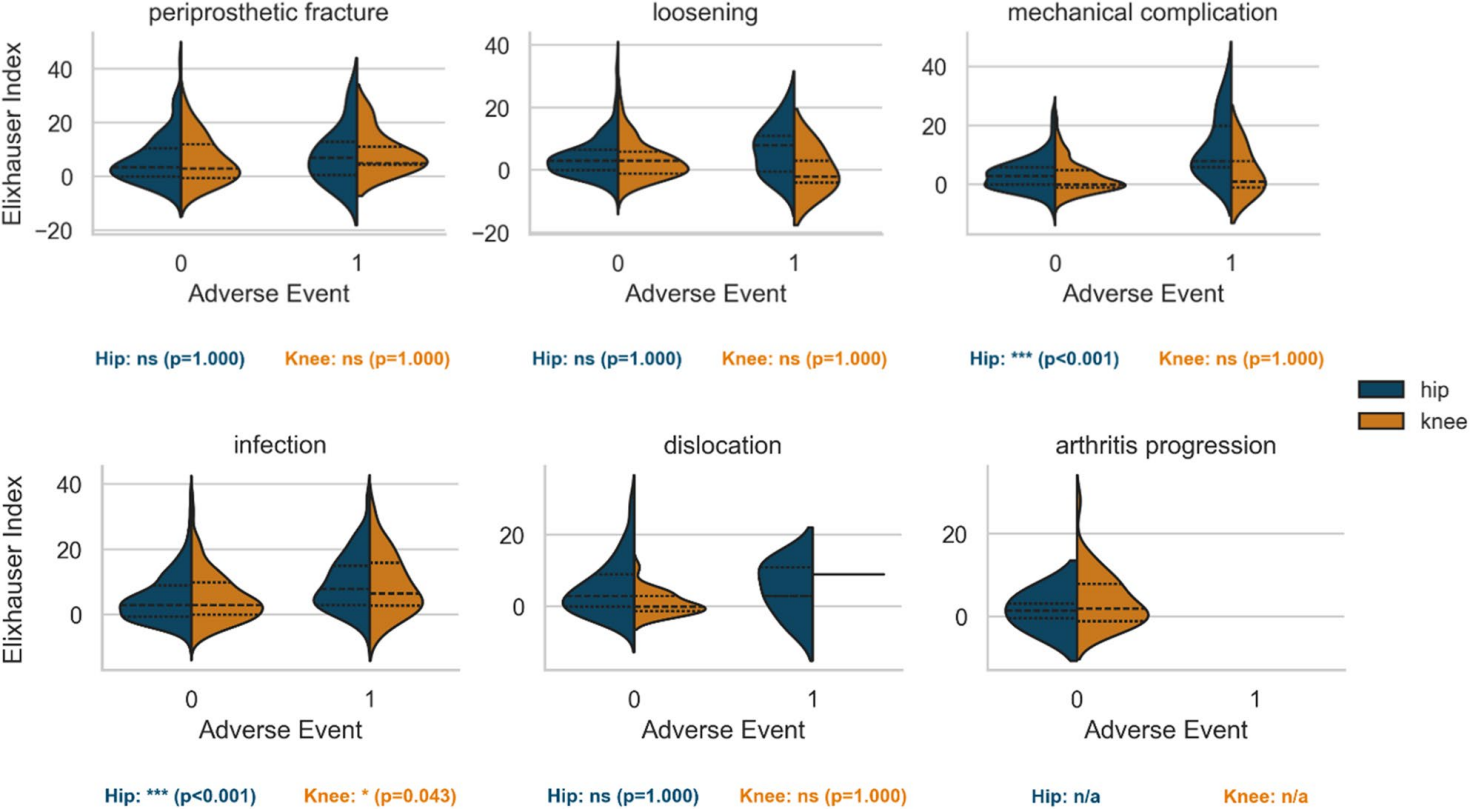
**Elixhauser Index stratified by joint**,** indication and Adverse Event.** AE – adverse event. Violin plots showing Elixhauser Index distribution by joint, indication, and AE status. Significant differences (*p* < 0.05) are marked (*)

**Fig. 4:**
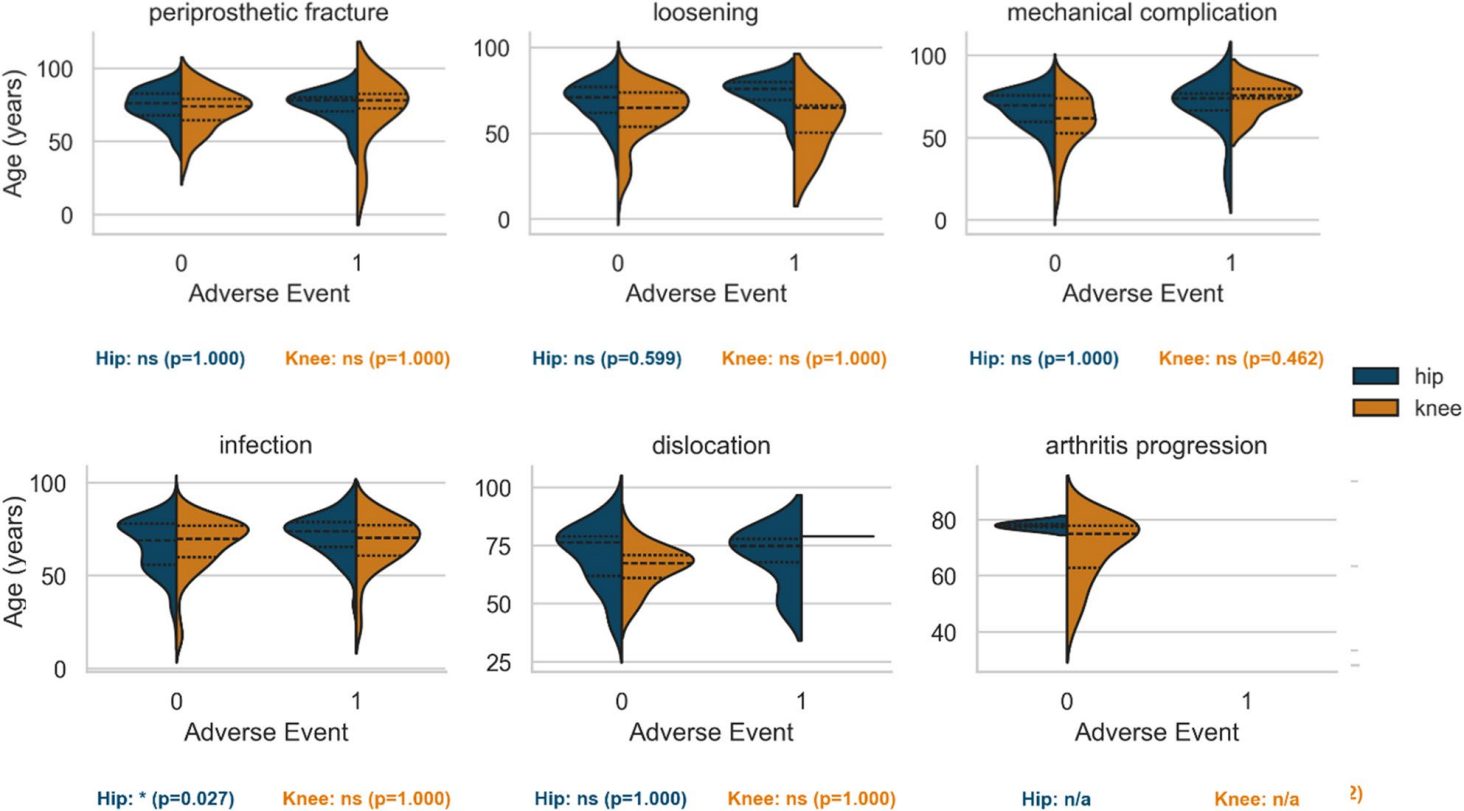
Age stratified by joint, indication and Adverse Event. AE – adverse event. Violin plot showing age distribution by joint, indication, and AE status. Significant differences (*p* < 0.05) are marked (*)

In rTHA, patients experiencing AE were significantly older (*p* = 0.002), an association not observed in rTKA. When stratified by indication, this age effect persisted only for rTHA revised for infection (*p* = 0.027; Fig. 3). The EI was significantly associated with AE occurrence in both hip and knee revisions (*p* < 0.001 for both). By indication, mechanical complication and infection remained significant predictors in rTHA (both *p* < 0.001), and infection was significant in rTKA (*p* = 0.043; Fig. 4).

Patients undergoing hip revision had significantly longer hospital stays than those undergoing knee revision (mean 19.5 ± 14.2 vs. 18.2 ± 13.6 days, *p* < 0.001). Older age was associated with increased LOS (*p* < 0.001), and this effect was observed in rTHA for infection, loosening, and mechanical complication, as well as in rTKA for loosening and mechanical complication (Appendix B).

Due to the data distribution, loosening was set as the reference group for multivariate analyses. In binary logistic regression, age, EI, joint, and the indications dislocation, infection, and periprosthetic fracture were identified as independent predictors of AE (AUC 0.76), with infection showing the strongest effect (OR 5.4, 95% CI 3.4–8.6, *p* < 0.001). Joint-specific models revealed that, in rTHA, periprosthetic fracture was not an independent predictor, whereas in rTKA, EI, infection, and periprosthetic fracture were significant, with periprosthetic fracture demonstrating the highest odds ratio (OR 9.8, 95% CI 2.3–41.1, *p* < 0.002; AUC 0.75 each).

**Fig. 5:**
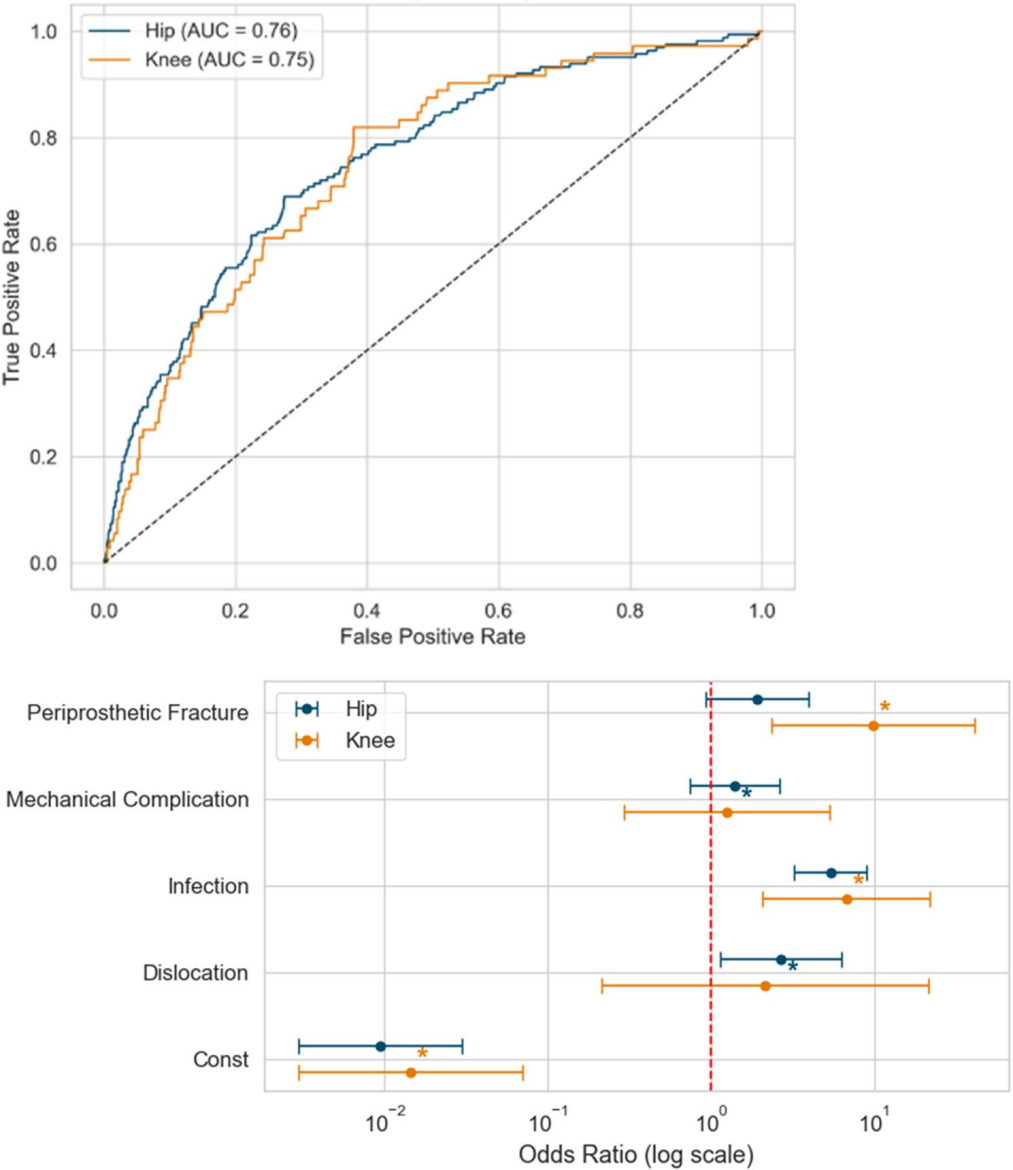
Comparison of Hip and Knee regression models on adverse event. ROC – receiver operating characteristic, AUC – area under the curve, AE – adverse event

**Fig. 6:**
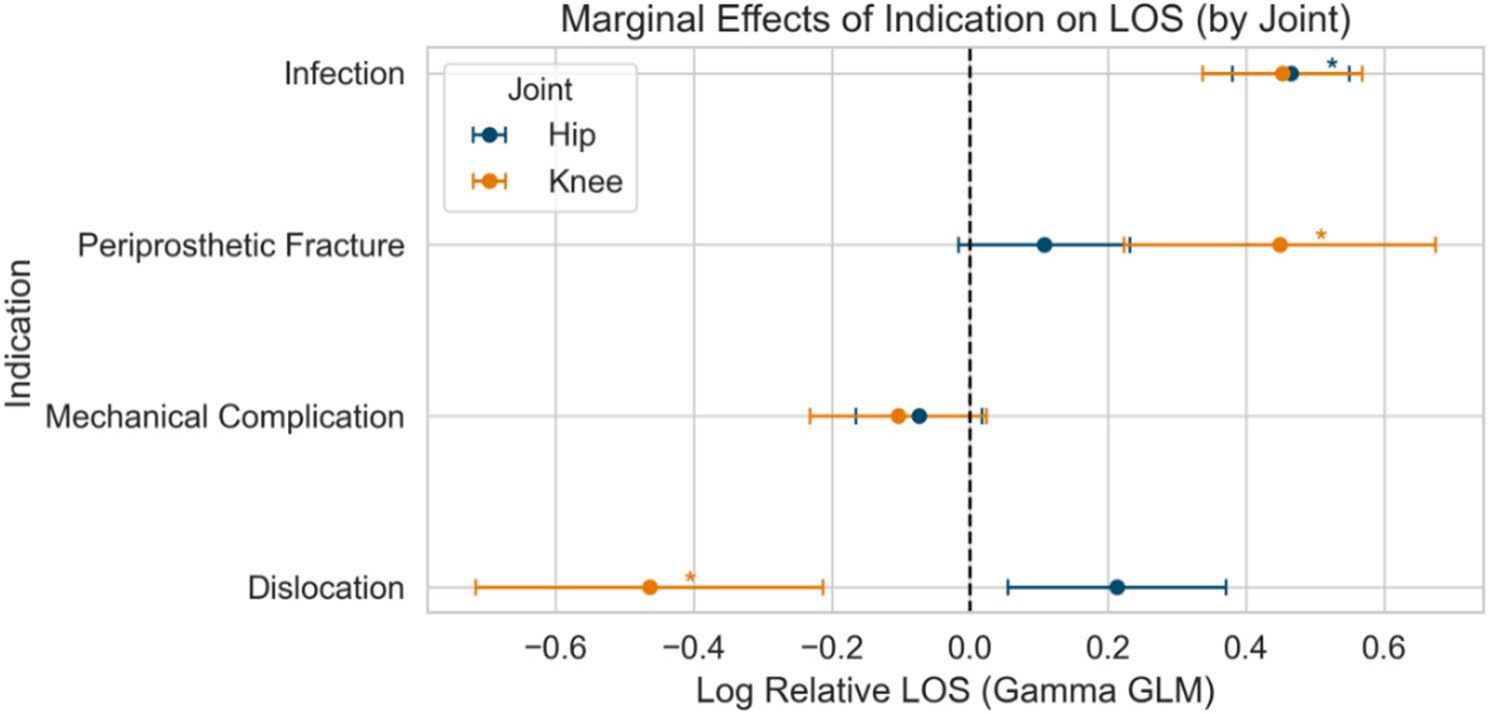
Comparison of Hip and Knee Generalised Linear Models on length of stay. LOS – length of stay. Forest plot comparing hip and knee marginal effects from Generalised Linear Models for predictors of hospital stay after revision arthroplasty. Regression coefficients with 95% confidence intervals are shown for joint and indication. Colored asterisks (*) indicate a significant association (*p* < 0.05) for the joint (hip: blue, knee: orange) with the greater effect

A generalised linear model identified age, EI, joint, infection, mechanical complication, and periprosthetic fracture as independent predictors of LOS, with infection again demonstrating the strongest association (coefficient 1.6, 95% CI 1.5–1.7). In rTHA, mechanical complication and periprosthetic fracture were not independently associated with LOS, while in rTKA, dislocation and mechanical complication were not significant predictors. Comparative analysis of regression coefficients between joints showed that periprosthetic fracture was a significantly stronger predictor of AE in knee revisions, while dislocation (in hip revisions) and periprosthetic fracture (in knee revisions) had a significant influence on LOS (Tables 2 and 3; Figs. 5 and 6).

**Table 2:** Adverse event model: coefficient differences (Hip vs. Knee)

	Hip		Knee		Comparison	
Variable	Coeff.	Std. err.	Coeff.	Std. Err.	Z (diff.)	*P* (diff)
Constant	-4.667	0.586	-4.24	0.81	-0.43	0.667
Age	0.019	0.008	0.00	0.01	1.40	0.162
Elixhauser Index	0.065	0.010	0.05	0.02	0.97	0.331
Dislocation	0.982	0.435	0.77	1.18	0.17	0.864
Infection	1.687	0.262	1.91	0.60	-0.34	0.734
Mechanical complication	0.334	0.327	0.23	0.74	0.13	0.897
**Periprosthetic fracture**	**0.652**	**0.370**	**2.28**	**0.73**	**-1.99**	**0.046**

Coeff. – Coefficient derived from logistic regression, Std. err. – standard error of the coefficient, Z (diff) – Z-statistics from the difference in coefficients, P (diff), p-value derived from the z-statistics. The table shows the statistical comparison of the logistic regressions on adverse event in hip – and knee revisions. Significant variables (p < 0.05) are highlighted in bold.

**Table 3:** Length of stay model: coefficient differences (Hip vs. Knee)

	Hip		Knee		Comparison	
Variable	Coeff.	Std. err.	Coeff.	Std. Err.	Z (diff.)	*P* (diff)
Constant	2.229	0.099	2.41	0.10	-1.32	0.186
Age	0.007	0.001	0.00	0.00	1.90	0.057
Elixhauser Index	0.013	0.002	0.01	0.00	0.79	0.429
**Dislocation**	**0.213**	**0.080**	**-0.46**	**0.13**	**4.47**	**0.000**
Infection	0.465	0.043	0.45	0.06	0.16	0.869
Mechanical complication	-0.074	0.046	-0.10	0.07	0.37	0.711
**Periprosthetic fracture**	**0.108**	**0.063**	**0.45**	**0.11**	**-2.60**	**0.009**

Coeff. – Coefficient derived from logistic regression, Std. err. – standard error of the coefficient, Z (diff) – Z-statistics from the difference in coefficients, P (diff), p-value derived from the z-statistics. The table shows the statistical comparison of the Generalised Linear Model on Length of Stay in hip – and knee revisions. Significant variables (p < 0.05) are highlighted in bold

Top: Side-by-side ROC curves comparing discriminative performance (AUC) of multivariate models for adverse event prediction in hip and knee revision arthroplasty.

Bottom: Forest plot comparing hip and knee logistic regression models for adverse event prediction after revision arthroplasty. Odds ratios with 95% confidence intervals are shown for joint and indication. Coloured asterisks (*) indicate a significant association (*p* < 0.05) for the joint (hip: blue, knee: orange) with the greater effect.

## Discussion

Revision arthroplasty procedures are inherently complex and associated with increased perioperative risk, longer hospital stay, and higher costs compared to primary procedures [1, 10], with a substantial increase of revision procedures projected [11, 12]. Our study examines known risk factors between revised joints and revision indications, aiming to identify “patients at risk” within a single tertiary care centre cohort to inform future risk stratification and resource allocation.

### Overall adverse event rates

The overall in-hospital AE rate observed in this study (13.1%) aligns well with previously reported rates for revision total joint arthroplasty, typically ranging from 4.7% to 10.6% [1, 3, 13, 14]. Consistent with prior literature, revision total hip arthroplasty (rTHA) demonstrated significantly higher AE rates compared to revision total knee arthroplasty (rTKA) [1, 13, 14]. Anatomical complexity, larger surgical exposures, proximity to critical neurovascular structures, and increased intraoperative blood loss in rTHA likely contribute to this increased risk [15, 16]. By contrast, certain aseptic rTKA procedures benefit from tourniquet use, limiting intraoperative blood loss, although prolonged operative times compared to rTHA can still contribute to risk [16].

### Risk factors of adverse events and length of hospital stay

The increased AE rate observed in rTHA was particularly pronounced for infections and mechanical complications, with additional trends towards higher rates observed in loosening and dislocation. Existing literature consistently identifies infection as the leading driver of perioperative risk, reporting 30-day complication rates of 7–10% after revision for periprosthetic joint infection [10, 17, 18]. Our study includes acute septic cases and patients with high comorbidity burden, which may explain why the AE rates are higher than those reported in the general literature. Comparable high postoperative AE rates have been documented at another German tertiary care centre among high-comorbidity rTHA patients, although indication-specific stratification was not provided [19]. Mechanical complications consistently demonstrate intermediate AE rates, whereas loosening and arthritis progression typically show lower, elective-level complication rates [14, 18]. Dislocation, while associated with moderate AE risk, notably carries high rates of recurrence [20].

Our data further identified a notably high AE rate for periprosthetic fractures in rTKA. This finding aligns closely with large registry analyses reporting 90-day mortality rates of 3.6–5.25%, among the highest across all revision indications [21, 22]. These fractures pose specific biomechanical challenges, particularly concerning tibial fractures near the patellar tendon insertion, which can require proximal tibial replacements and/or extensive soft tissue reconstructions, significantly increasing perioperative complexity [23].

The mean LOS in our cohort (19 ± 14 days) substantially exceeds internationally reported durations of 2–6 days for revision arthroplasty [10, 11, 17, 24–27]. Extended LOS likely reflects system-level factors unique to Germany, including differing admission thresholds, discharge protocols, and reimbursement structures rather than differences in clinical effectiveness or patient risk profiles [28, 29]. German tertiary care centres further report prolonged hospital stays which they primarily associate with higher patient comorbidity burdens and poorer preoperative physical health rather than system-level differences alone [19].

Patient-level risk factors, particularly advanced age and elevated Elixhauser Index (EI), significantly influenced perioperative outcomes in this study. Advanced age was specifically associated with increased AE risk in rTHA infections, aligning with literature indicating that patients aged ≥ 80 years have higher risks of medical complications and mortality after revision arthroplasty, particularly in rTHA [4, 30, 31]. Similarly, elevated EI consistently predicted both AE and LOS across joints and multiple surgical indications, confirming its strong discriminative ability for perioperative risk stratification [4, 19]. Additional modifiable risk factors, such as smoking and elevated BMI, have also been associated with extended hospital stays and increased complication risks [26], but are not available in the present study.

### Independent predictors and patients at risk

Our multivariate analyses identified increasing age, elevated EI, joint type (hip), and specific surgical indications (infection, dislocation, periprosthetic fracture) as independent predictors of AE (AUC 0.76). Infection notably emerged as the strongest predictor of adverse outcomes (OR 5.4), which is consistent with evidence from large datasets demonstrating significantly increased complication rates, prolonged LOS and mortality for periprosthetic joint infection [32–34].

Joint-specific analyses refined these predictive relationships further. In rTHA, age, EI, infection, and dislocation independently predicted AE risk, whereas periprosthetic fracture did not. Conversely, in rTKA, EI, infection, and particularly periprosthetic fracture (OR 9.8) significantly predicted AE. These findings align with previous studies emphasising the severe complication and mortality risk specifically associated with periprosthetic fractures in revision knee arthroplasty [2, 21, 35].

LOS predictors included age, EI, joint type (hip), infection, mechanical complications, and periprosthetic fracture, with infection showing the strongest predictive association (coefficient 1.6). Joint-specific analyses highlighted unique relationships, with dislocation significantly influencing LOS in rTHA, and periprosthetic fracture markedly impacting LOS in rTKA. Literature consistently supports these joint-specific differences, confirming higher AE rates and longer LOS for rTHA relative to rTKA, even after controlling for patient demographics and surgical indication [36, 37].

Advanced age consistently emerged as a significant independent predictor of AE and LOS, particularly notable in infection-related rTHA revisions. Existing literature robustly supports increased risks for patients aged ≥ 80 years, particularly regarding medical complications and mortality after revision arthroplasty [4, 31, 38]. Additionally, EI demonstrated superior discriminative capacity over age alone for perioperative risk assessment, providing a comprehensive evaluation of patient complexity to guide perioperative care strategies [4, 7, 19].

Despite structured preoperative multidisciplinary assessment and optimisation strategies at our tertiary care institution, observed outcomes indicate these measures alone do not sufficiently mitigate risks in patients presenting with severe surgical indications or high comorbidity burdens. While this has also been found by other German tertiary care centres, it underscores the importance of targeted, individualised perioperative care pathways and structured resource planning, particularly addressing infection cases, periprosthetic fractures in knees, and elderly patients with significant comorbidities. These could include enhanced perioperative resource allocation, focused ICU management and structured discharge planning optimizing resource allocation, which are the subject of future studies.

## Strengths and limitations

This study involves a large sample size, resulting in a robust dataset spanning a decade. The data were meticulously cleaned, structured, and categorised to provide detailed insights. Clinically relevant endpoints were selected, and the comorbidity index applied is both widely recognised and validated. The cohort is diverse, covering a broad range of indications and patient profiles. However, several limitations are present. The retrospective design may introduce selection and information biases. These are addressed by not applying inclusion or exclusion criteria, except for indications comprising < 1% of the cohort. Reporting all cases of hardware prosthesis revisions within the study period, the dataset included a small paediatric population which is rare in arthroplasty revision. Being a single-centre study limits the generalisability of the findings to other similarly large and specialised centres, and causality is precluded due to the study design. Data for LOS were missing in 3% of cases, which may introduce selection bias. Finally, confounding by unmeasured variables, such as preoperative functional status or procedural complexity, cannot be entirely excluded.

## Conclusion

This study demonstrates that the incidence of perioperative adverse clinical outcomes is substantial in revision arthroplasty. Infection (in all revisions), periprosthetic fractures (in knee revisions), advanced age, and high comorbidity burden were identified as critical independent predictors of adverse events and prolonged hospitalisation. Despite systematic preoperative optimisation, these factors significantly influence patient outcomes, underscoring the necessity of targeted, individualised perioperative care pathways. Future studies should include risk-stratification tools and examine potential targets such as enhanced perioperative resource allocation, focused ICU management and structured discharge planning.

## Supplementary Information

Below is the link to the electronic supplementary material.

Supplementary Material 1

Supplementary Material 2

Supplementary Material 3

## Data Availability

All relevant data and findings are included in the manuscript, additional data are available from the corresponding author upon reasonable request.

## Abbreviations

AE: adverse event
AUC: area under the curve
CI: confidence interval
Coeff: coefficient
EC: Elixhauser Comorbidity/Comorbidities
EI: Elixhauser Index
ICD: International Classification of Diseases; ICD-10-GM 10th Revision, German Modification
ICU: intensive care unit
IQR: interquartile range
LOS: length of stay
OPS: Operationen- und Prozedurenschlüssel (German adaptation of ICD-10-PCS)
OR: odds ratio
PCS: Procedure Coding System
rTHA: revision total hip arthroplasty
rTKA: revision total knee arthroplasty
ROC: receiver operating characteristic
SD: standard deviation
