# Supplementary material for: Identifying patients at risk in revision arthroplasty: a comprehensive single-centre analysis
Source: J Orthop Surg Res. 2026 Jan 15;21:54. doi: 10.1186/s13018-025-06625-y (PMC12849725; doi:10.1186/s13018-025-06625-y)
Supplement: Supplementary file 1 — Supplementary Material 1 [file 13018_2025_6625_MOESM1_ESM.pdf]

Appendix A – Systematic Adverse Event Assessment

Category	ICD_Codes
Symptoms and signs involving the digestive system and abdomen	R10.0, R10.1, R10.2, R10.3, R10.4
Vascular disorders of the intestine	K55.0, K55.1, K55.21, K55.22, K55.31, K55.8
Complications of surgical and medical care, not elsewhere classified	T81.0, T81.1, T81.2, T81.3, T81.4, T81.5, T81.6, T81.7, T81.8, T81.9
Pyogenic arthritis	M00.00, M00.01, M00.02, M00.03, M00.04, M00.05, M00.06, M00.07, M00.08, M00.09, M00.10, M00.11, M00.12, M00.13, M00.14, M00.15, M00.16, M00.17, M00.18, M00.19, M00.20, M00.21, M00.22, M00.23, M00.24, M00.25, M00.26, M00.27, M00.28, M00.29, M00.80, M00.81, M00.82, M00.83, M00.84, M00.85, M00.86, M00.87, M00.88, M00.89, M00.90, M00.91, M00.92, M00.93, M00.94, M00.95, M00.96, M00.97, M00.98, M00.99
Cutaneous abscess, furuncle and carbuncle	L02.0, L02.1, L02.2, L02.3, L02.4, L02.8, L02.9
Internal derangement of the knee	M23.50, M23.51, M23.52, M23.53, M23.54, M23.57, M23.59, M23.60, M23.61, M23.62, M23.63, M23.64, M23.67, M23.69, M23.80, M23.81, M23.82, M23.83, M23.84, M23.87, M23.89, M23.90, M23.91, M23.92, M23.93, M23.94, M23.95, M23.96, M23.97, M23.99
Complications of internal orthopedic prosthetic devices, implants and grafts	T84.00, T84.01, T84.02, T84.03, T84.04, T84.05, T84.06, T84.07, T84.08, T84.09, T84.10, T84.11, T84.12, T84.13, T84.14, T84.15, T84.16, T84.18, T84.20, T84.28, T84.3, T84.4, T84.5, T84.6, T84.7, T84.8, T84.9
Other disorders of the muscle	M62.40, M62.41, M62.42, M62.43, M62.44, M62.45, M62.46, M62.47, M62.48, M62.49
Phlebitis and thrombophlebitis	I80.0, I80.1, I80.20, I80.28, I80.3, I80.80, I80.81, I80.88, I80.9

To begin the analysis, an initial review of the data to identify the different complications present within the dataset was conducted - this process involved systematically filtering and categorising all available secondary diagnosis of the dataset, stepwise searching for specific ICD codes associated with various medical complications, allowing to determine which types of complications were recorded – and how.

Following this, the data was scanned for potential subcategories within these complications. Subcategories were identified based on more detailed aspects of the complications -including its clinical relevance- and were then used to conduct a more in-depth statistical analysis, resulting in the structure provided in table 1 of the manuscript. This step ensured that the analysis captured both the broader categories and the specific nuances within each complication type associated with orthopaedic surgery. Using this method around 15000 possible ICD/OPS codes were narrowed down to 197 codes that were present within the dataset and defined as AE, leading to the following systematic.

Feature	Codes (ICD/OPS)
AE_ICD_abd_acute	R10.0
AE_ICD_abd_vessel_occlusion	K55.0
AE_ICD_bleeding_OP	T81.1
AE_ICD_foreignbody_OP	T81.5
AE_ICD_haematoma_bleeding_OP	T81.0
AE_ICD_wound_OP	T81.2
AE_ICD_inf_OP	T81.4
AE_ICD_periop_other_OP	T81.8, T81.9
AE_ICD_periop_vasc_OP	T81.7
AE_ICD_TVT	I80.0, I80.1, I80.20, I80.28, I80.3, I80.80, I80.81, I80.88, I80.9
AE_ICD_woundhealing_OP	T81.3
AE_ICD_abd_ileus	K56.0, K56.3, K56.5
AE_ICD_abd_ileus_other	K56.6, K56.7, K56.4
AE_ICD_ROSC	I46.0
AE_ICD_Death	I46.1, I46.9
AE_Cerebralhemmorrhage	I60., I60.0, I60.1, I60.2, I60.3, I60.4, I60.5, I60.6, I60.7, I60.8, I60.9, I61., I61.0, I61.1, I61.2, I61.3, I61.4, I61.5, I61.6, I61.8, I61.9, I62., I62.0, I62.00, I62.01, I62.02, I62.09, I62.1, I62.9
AE_ICD_PE	I26.0, I26.9
AE_ICD_Myokardinfarkt	I21.0, I21.1, I21.2, I21.3, I21.4, I21.40, I21.41, I21.42, I21.48, I21.9
AE_Renalinsufficiency_acute	N17.0, N17.01, N17.02, N17.03, N17.09, N17.1, N17.11, N17.12, N17.13, N17.19, N17.2, N17.21, N17.22, N17.23, N17.29, N17.8, N17.81, N17.82, N17.83, N17.89, N17.9, N17.91, N17.92, N17.93, N17.99
AE_ICD_Stroke	I63., I63.0, I63.1, I63.2, I63.3, I63.4, I63.5, I63.6, I63.8, I63.9, I64
AE_OP_abd_laparotomy	5-541.0, 5-541.1, 5-541.2, 5-541.3, 5-541.4, 5-541.5, 5-541.6, 5-541.x, 5-541.y
AE_OP_vasc_surgical_,abdpelvother	5-389.5x
AE_OP_vasc_surgical_,AKneeOther	5-389.7x
AE_OP_vasc_surgical_,Aproffem	5-389.71
AE_OP_vasc_surgical_,vascother	5-389.ax, 5-389.x, 5-389.y
AE_OP_Vesselreconstruction_other	5-388.70, 5-388.9B, 5-395.70, 5-394.1
AE_OP_closureartery	5-389.5X, 5-389.71, 5-389.7X, 5-389.X
AE_OP_closurevein	5-389.A5, 5-389.9K
AE_ICD_CPR	8-779
