# Supplementary material for: Identifying patients at risk in revision arthroplasty: a comprehensive single-centre analysis
Source: J Orthop Surg Res. 2026 Jan 15;21:54. doi: 10.1186/s13018-025-06625-y (PMC12849725; doi:10.1186/s13018-025-06625-y)
Supplement: Supplementary file 2 — Supplementary Material 2 [file 13018_2025_6625_MOESM2_ESM.pdf]

# Appendix B – Distribution of Length of Stay across age and indication

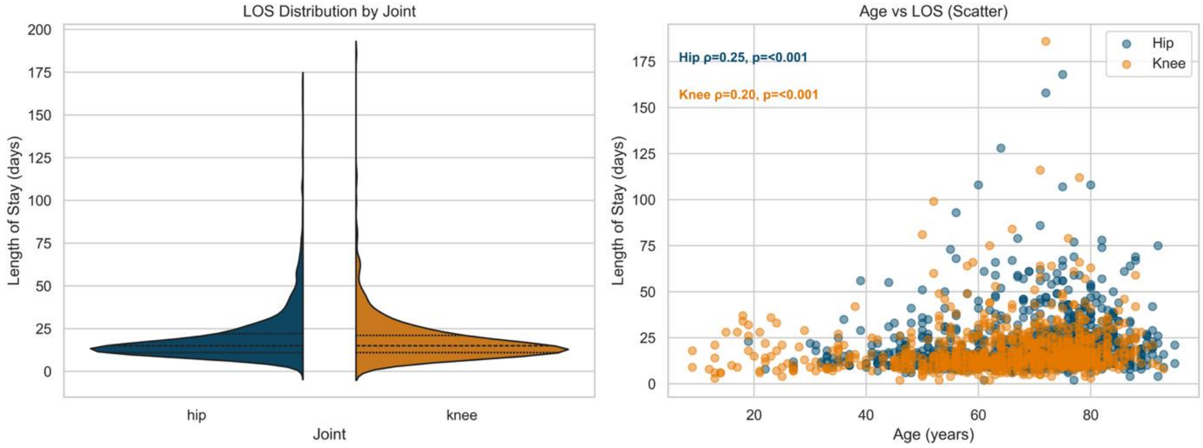


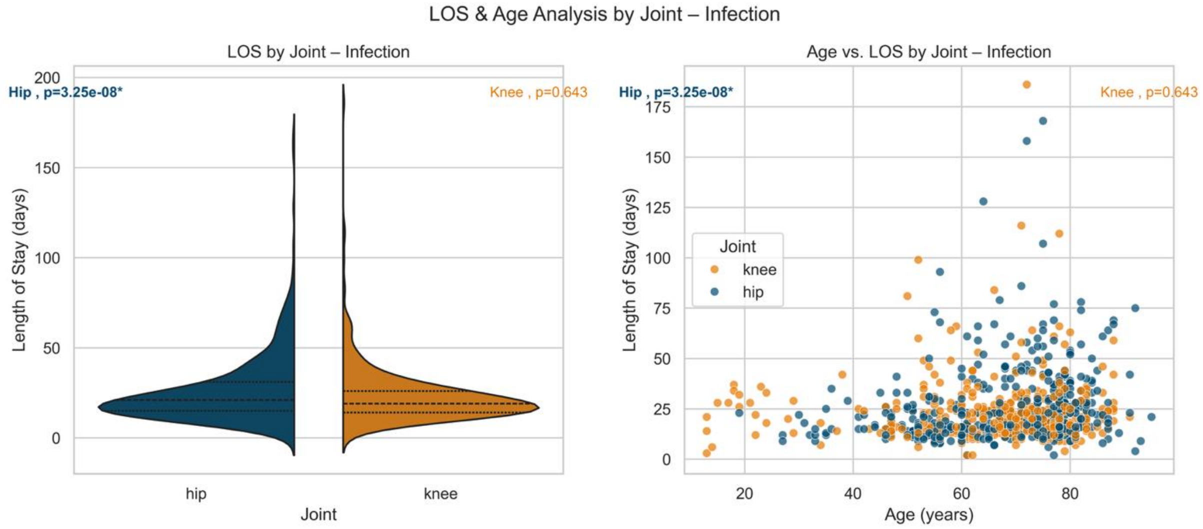

LOS & Age Analysis by Joint – Mechanical complication

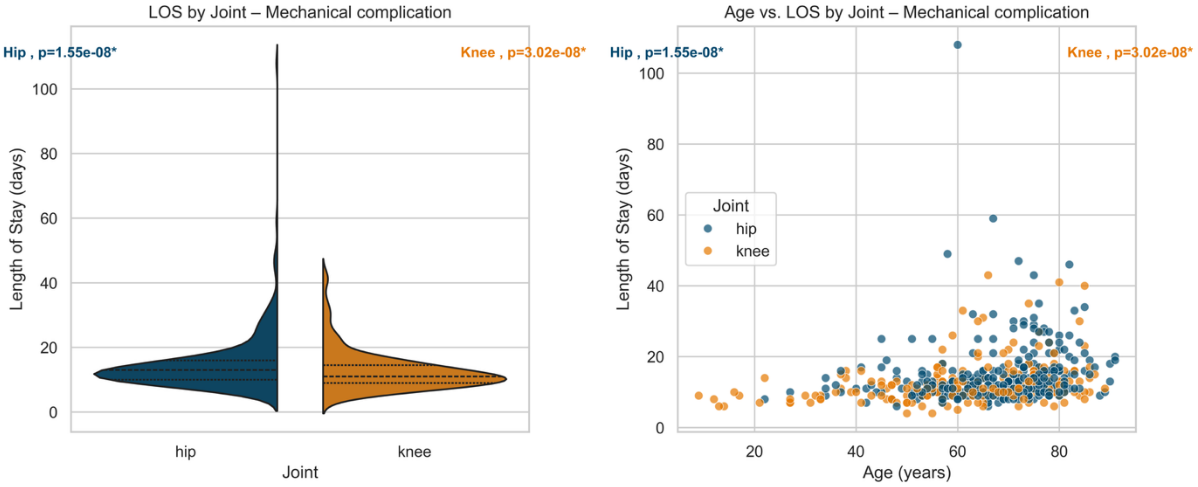


LOS & Age Analysis by Joint – Periprosthetic fracture

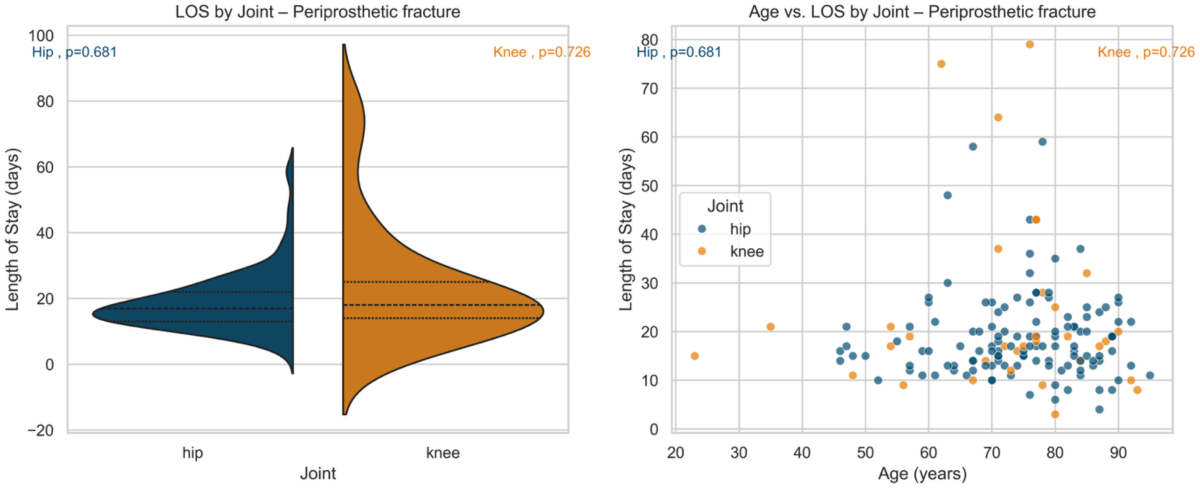

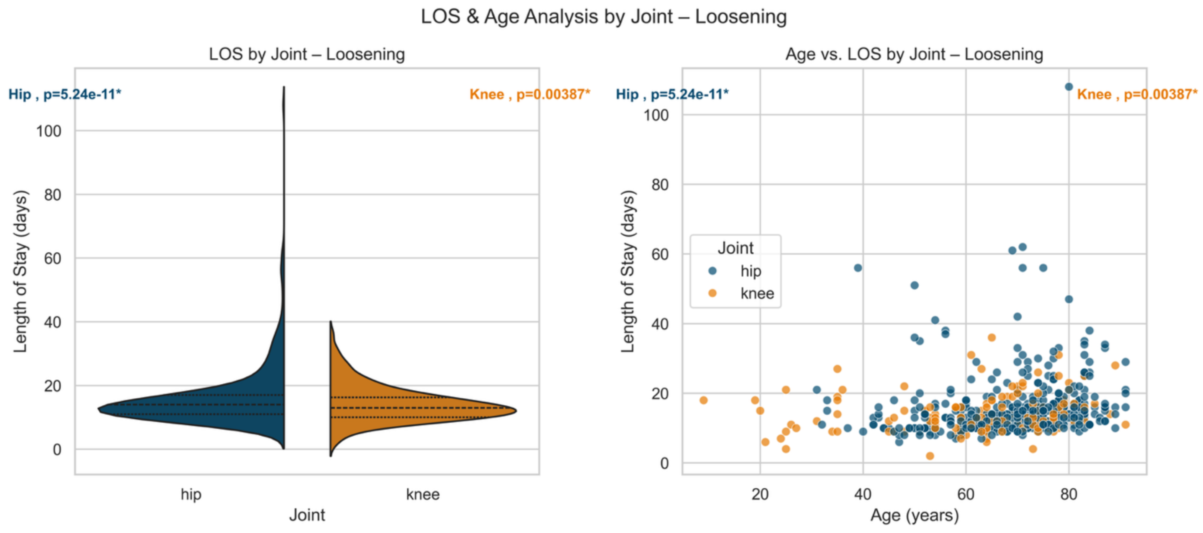
LOS & Age Analysis by Joint – Loosening
LOS by Joint – Loosening
Hip , p=5.24e-11*
Knee , p=0.00387*
Length of Stay (days)
0
20
40
60
80
100
hip
knee
Joint
Age vs. LOS by Joint – Loosening
Joint
hip
knee
20
40
60
80
Age (years)

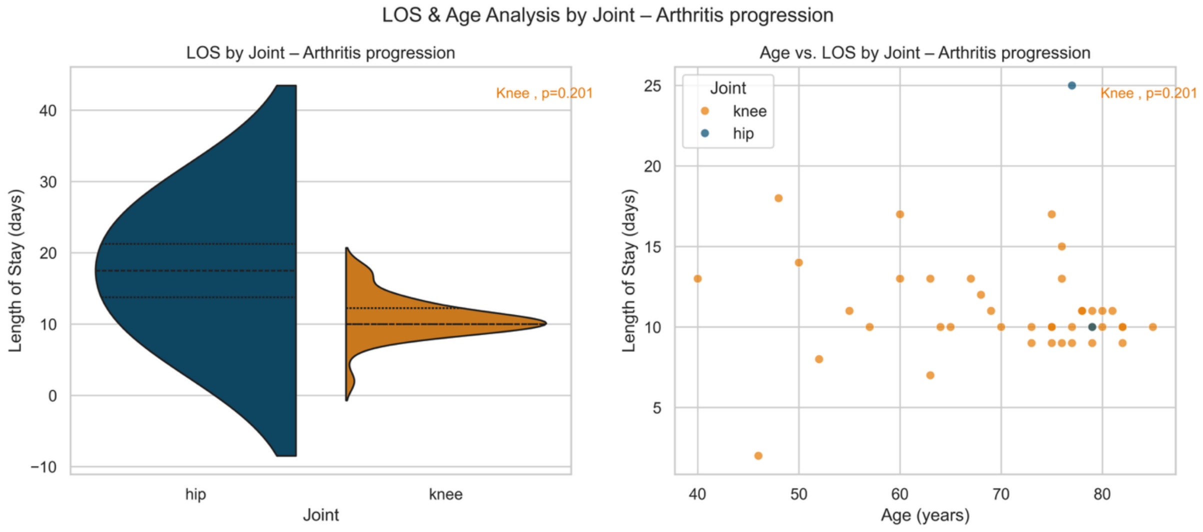
LOS & Age Analysis by Joint – Arthritis progression
LOS by Joint – Arthritis progression
Knee , p=0.201
Length of Stay (days)
−10
0
10
20
30
40
hip
knee
Joint
Age vs. LOS by Joint – Arthritis progression
Joint
knee
hip
5
10
15
20
25
40
50
60
70
80
Age (years)

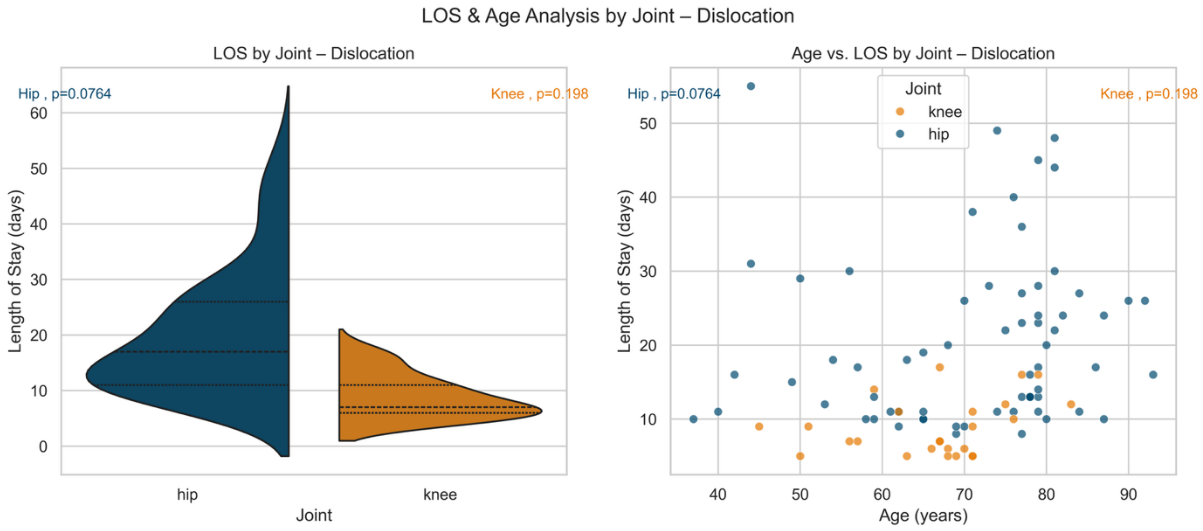
LOS & Age Analysis by Joint – Dislocation
LOS by Joint – Dislocation
Hip , p=0.0764
Knee , p=0.198
Length of Stay (days)
0
10
20
30
40
50
60
hip
knee
Joint
Age vs. LOS by Joint – Dislocation
Joint
knee
hip
10
20
30
40
50
40
50
60
70
80
90
Age (years)
