# Supplementary material for: Identifying patients at risk in revision arthroplasty: a comprehensive single-centre analysis
Source: J Orthop Surg Res. 2026 Jan 15;21:54. doi: 10.1186/s13018-025-06625-y (PMC12849725; doi:10.1186/s13018-025-06625-y)
Supplement: Supplementary file 3 — Supplementary Material 3 [file 13018_2025_6625_MOESM3_ESM.pdf]

Appendix C – Adverse Events across Revision Indications

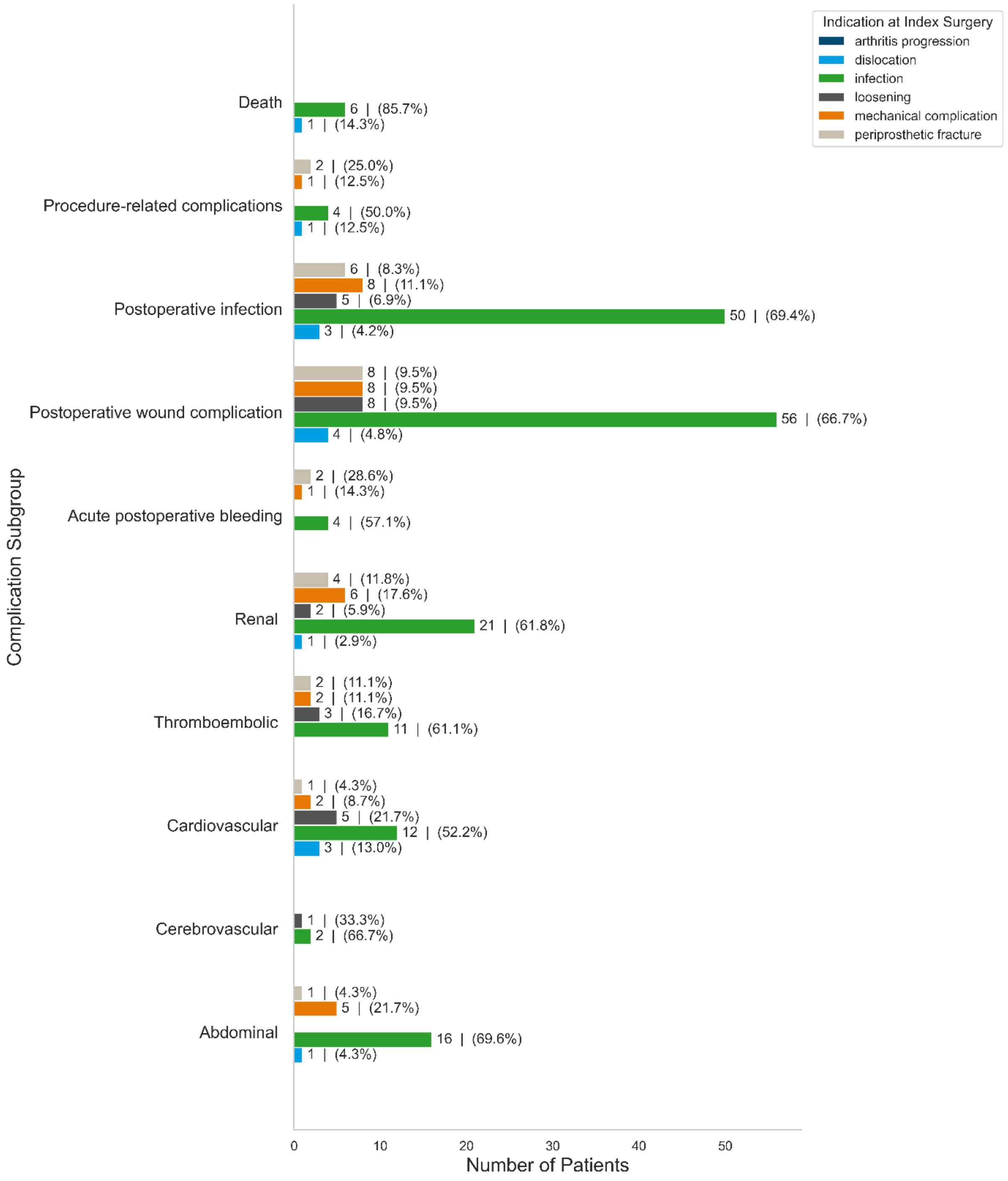

Grouped bar plot showing the number and percentage of patients with each complication subgroup, stratified by indication at index surgery. Each bar represents one indication, colored according to the legend. Counts and percentages within each subgroup are shown at the end of each bar.
